# Integrated assessment of emerging science and technologies as creating learning processes among assessment communities

**DOI:** 10.1186/s40504-016-0042-6

**Published:** 2016-07-28

**Authors:** Ellen-Marie Forsberg, Barbara Ribeiro, Nils B. Heyen, Rasmus Øjvind Nielsen, Erik Thorstensen, Erik de Bakker, Lars Klüver, Thomas Reiss, Volkert Beekman, Kate Millar

**Affiliations:** 1 Research Group on Responsible Innovation, Oslo and Akershus University College, P.O. Box 4 St Olavs plass, 0130 Oslo, Norway; 2 Centre for Applied Bioethics, University of Nottingham, Sutton Bonington, Leicestershire, LE12 5RD UK; 3 Fraunhofer Institute for Systems and Innovation Research ISI, Breslauer Strasse 48, 76139 Karlsruhe, Germany; 4 Danish Board of Technology, Toldbodgade 12, 1253 Copenhagen, Denmark; 5 Agricultural Economics Research Institute (LEI), Wageningen University and Research Centre, P.O. Box 29703, 2502LS ‘S Gravenhage, The Netherlands

**Keywords:** Emerging science and technologies, Assessment, Dialogue, Integration, Transparency, TranSTEP

## Abstract

Emerging science and technologies are often characterised by complexity, uncertainty and controversy. Regulation and governance of such scientific and technological developments needs to build on knowledge and evidence that reflect this complicated situation. This insight is sometimes formulated as a call for integrated assessment of emerging science and technologies, and such a call is analysed in this article. The article addresses two overall questions. The first is: to what extent are emerging science and technologies currently assessed in an integrated way. The second is: if there appears to be a need for further integration, what should such integration consist in? In the article we briefly outline the pedigree of the term ‘integrated assessment’ and present a number of interpretations of the concept that are useful for informing current analyses and discussions of integration in assessment. Based on four case studies of assessment of emerging science and technologies, studies of assessment traditions, literature analysis and dialogues with assessment professionals, currently under-developed integration dimensions are identified. It is suggested how these dimensions can be addressed in a practical approach to assessment where representatives of different assessment communities and stakeholders are involved. We call this approach the Trans Domain Technology Evaluation Process (TranSTEP).

## Introduction

Integrated approaches to the assessment of technology and policy choices are found in several assessment traditions. Historically, integrated approaches have been considered as particularly appropriate for assessing complex systems that are in danger of being reduced to their composite parts, and have as such been a subject of study within systems thinking (see e.g. Smith 2010). An important motivation for developing integrated approaches has been to avoid reducing decisions with important social and environmental implications to an economic issue and such approaches have arguably been especially explored in the field of sustainability assessment, where practitioners have formed The Integrated Assessment Society (TIAS).

Recognising sustainability as a key goal of environmental management reinforces the significance of non-fragmentation and non-reduction (Bond et al. 2012). A wide range of researchers working on environmental management have contributed with important work on developing non-reductive integrated assessments over the last few decades (see for instance de Ridder et al. 2007, Van der Sluijs 2002 and Van Asselt et al. 2001). Some of these approaches are based on computational simulation models (e.g. Epstein 1999 and Hare and Deadman 2004), while others have followed a more deliberative approach (Soncini-Sessa et al. 2007 and Cohen and Neale 2006).

In the context of sustainability assessment approaches, Van der Sluijs (2002) provides the following definition for the term:

Integrated assessment (IA) is a reflective and iterative participatory process that links knowledge (science) and action (policy) regarding complex global change issues such as acidification and climate change. IA can be defined as an interdisciplinary process of combining, interpreting and communicating knowledge from diverse scientific disciplines in such a way that the whole cause–effect chain of a problem can be evaluated from a synoptic perspective with two characteristics: (i) it should have added value compared to single disciplinary assessment; and (ii) it should provide useful information to decision makers (Rotmans and Dowlatabadi, 1998).

However, the concept of integrated assessment can be understood in various ways. Technology Assessment (TA) is another important assessment tradition that has regarded itself as having an integrating function, although the term ‘integrated assessment’ has not been a prominent concept. TA developed from decades of debate on the impacts and governance of science and technology (S&T), especially nurtured by post-war science and technology studies (STS) (van den Ende et al. 1998), and has revolved around exploring the relation between science, technology and society, including policy-making. In parallel to a critique on the limitations of expert advice and scientific reasoning in controversial and politicised science and technology issues (Wynne 1992, Jasanoff 2003), TA institutions and bodies of practice were encouraged to open up their processes to a plurality of actors and to adopt a more constructivist approach to technology assessment (e.g. Schot and Rip 1997, Guston and Sarewitz 2002). TA has therefore played a significant role in the development of participatory methods for democratic deliberation on policies dealing with the future options and risks of science and technology development (Joss and Bellucci 2002).

Other assessment traditions, or ‘advisory domains’ as we will refer to them in the following,^1^ also have specific integrated approaches. For instance, impact assessments (IA), as used by the European Commission, have an integrated character where the emphasis is on causal analysis of the effects of policy interventions.^2^ Also risk management has integrated approaches, such as integrated risk-benefit assessment.^3^

Emerging science and technologies (EST) appear prima facie to be in need of integrated assessment because they are often characterised by complexity, uncertainty and controversy with regard to facts and values. Emerging science and technologies are not neatly defined, but the term is usually restricted to technologies ‘that are at their early stage of development at a science and technology level’ (EC 2006, p. 13), and often includes biotechnologies, nanotechnologies, neurotechnologies and ICTs (see e.g. Robinson et al. 2013). Prima facie, arguments for integration in assessments of emerging science and technologies are related to their complex nature, having potentially significant, but to varying extents uncertain, effects in environmental, economic and social systems. Their emergent nature indicates that the uncertainties around them potentially display unknown complexity and are thus urgent to address. A reductionist or fragmented evidence base for policy- and decision-making in this field may have significant medium and long term impacts with regard to health, the environment and the economy.

Despite this situation and despite the broad variety of existing integrated approaches, there is scarce knowledge of the extent to which such technologies are being assessed in integrated ways. The questions addressed in this article are therefore to what extent emerging science and technologies are being assessed in an integrated way and, if there appears to be a need for further integration, what such integration should consist in.

We will start with a brief presentation of the methods applied in this research (Methods) and a presentation of the basis for our analysis of integration in this article (Identifying the key integration dimensions). We will then present the main findings from four case studies of assessment of specific emerging technologies analysed against this basis (Findings from the analysis of case studies). Based on the analysis of integration dimensions in the case studies, on literature studies and on dialogues with assessment professionals we will then identify integration dimensions that are currently under-developed (What kind of integration should be strengthened in EST assessment?). As a response to this gap we describe and justify an approach for further integration (The Trans Domain Technology Evaluation Process (TranSTEP)). After a discussion of the assumptions of this work and the novelty of the suggested approach (Discussion), we conclude the paper with a summary of the main points (Conclusion).

## Methods

Four case studies of EST assessment will here be presented: nano food in the Netherlands, synthetic biology in Germany, biofuels in the UK and cloud computing in Denmark (see De Bakker et al. 2014, van Doren and Heyen 2014, and Boucher et al. 2014). Relevant European level assessments and policy instruments were also included in these studies. In addition, analyses of different advisory domains are presented to contextualise the findings in the case studies (see Forsberg et al. 2014). The case and domain studies included screening 1506 assessments and reviewing 101 assessments with an analytical approach presented in detail in Forsberg et al. 2014. The studies were conducted in the European Commission FP7 EST-Frame project,^4^ in the period of 2012 to 2014.

The analytic protocol of the case and domain studies included two tables; a purpose analysis table (developed in Decker and Ladikas 2004) and a process characterisation table. The purpose analysis table was used to map whether the individual assessment intended to raise knowledge, form attitudes or initialise action and whether it focused on scientific/technological, societal or policy aspects. The process characterisation table was used to map the process characteristics of the assessments, such as the participation in the assessment, the kind of impacts considered, the transparency, the focus on values and the evidence base. These tables were used to score each reviewed assessment. A calibration group (consisting of researchers from the EST-frame project team) developed a guidance document in order to help standardise the reviews and engaged in dialogue about correct scoring practices across the evaluators. The results were aggregated on the case study level and on the domain study level.

Each case study included an explicit review of the current state of integration in the assessments and potential further needs for integration. In addition, integration was discussed with assessment practitioners, policy makers and stakeholders in case study workshops, in a broader assessment practitioner workshop, with the project’s advisory committee and at conferences. For more detail on the method and results of these studies please see the above mentioned publications. The purpose of this article is not to discuss the studies in detail, but to reflect on the overall findings.

The results of these analytical and empirical studies, literature studies and dialogues with assessment professionals, policy makers and stakeholders, were the basis for developing an approach to integrated assessment that would address the observed under-developed aspects of integration. Elements of this approach, initially called the Integrated EST Framework, was applied in four ‘testing workshops’ (corresponding to the four technology case fields) and discussed with European civil servants in another workshop. Subsequently it was finalised into the so-called TranSTEP approach, the implementation of which was discussed with end users. The end-users in the project included assessment professionals from different domains, scientists, philosophers, representatives from industry and other stakeholders, and civil servants from national and European institutions.

### Identifying the key integration dimensions

Even if integrated assessment has been called for by some policy makers (see European Commission 2010), this has not reflected an unequivocal call from a broad range of assessment practitioners. This can be partially explained by the fact that integration in assessment has been haunted by a lack of concise terminology (Scrase and Sheate 2002). Although the notion of integrated assessment is firmly established in policy (such as in the integrated approach of European Impact Assessments), its meaning is not singularly defined. In order to analyse the need for more integrated EST assessment we will therefore here spell out different interpretations or dimensions of integrated assessment (see also Forsberg and de Lauwere 2012).

Scholars have earlier discussed different understandings of integration and integrated assessment. In the sustainability assessment context Scrase and Sheate (2002) identified 14 different meanings of ‘integrated’ related to ‘integrated assessments’ in environmental governance. These include issues such as better coordination and dissemination of data; inclusion of specific environmental concerns into governance; better coordination between high level and more local level governance; not isolating specific environmental problems at the cost of the whole; life cycle analysis; integration of business concerns into governance; integration of the three pillars of sustainability into governance; integration across policy domains; integrated computer modelling; integration of other stakeholders into governance; integration among assessment tools; integration of equity concerns into governance; and proper integration of assessment into governance.

Some of these have prima facie relevance also for the assessment of emerging science and technologies specifically. However, assessment of emerging technologies raises some particular challenges that may not be equally relevant in the sustainability assessment tradition. These concern issues like the uncertainty and controversy of facts and values related to the technology, how to tackle ESTs’ potential to challenge our concepts of natural/artificial, human/machine or identity, the accelerating speed of technological change, and the possibility of adequate governance in a globalised market economy. Prima facie, integrated EST assessment might need to somehow incorporate such characteristics and a revised version of Scrase and Sheate’s list, adapted to issues central to EST governance, has been developed. The following list with interpretations of the notion of integration in EST assessment has proved useful for our analyses:

- **Inclusion of all areas of topics into assessments**: This understanding of integration refers to inclusion of a broad scope of issues into assessments in a given assessment domain, such as an ethical assessment dealing with human rights, animal welfare, environmental integrity, global justice, individual autonomy, privacy, security aspects, etc. related to a specific technology.
- **Inclusion of values into assessments**: This understanding refers to specific deliberation on ethical assumptions and normative stances in the assessments based on the observation that assessments often make normative assumptions that affect the assessment conclusions but do not reflect on these (see. e.g. Mongin 2006).
- **Inclusion of narratives, visions or worldviews into assessments**: Such an understanding of integration is based on arguments, in particular from the European DEEPEN project (Davies et al. 2009), where it was pointed out that narratives are likely to influence perceptions and evaluations of technologies and that as such they need reflection. This is related to the dimension of inclusion of values above, but more specifically directed towards lay ethics.
- **Not isolating one topic at the expense of the whole**: This holistic perspective is called for, for instance expressed in the Science-in-Society work programme for 2011 (EC 2011), in the analysis that partial assessments are not sufficient for aligning EST with societal demands.
- **Explicating assessment framing**: By reference to the TAMI project in the TA domain (Decker and Ladikas 2004) explicit situation analysis and framing of assessments have been argued to be essential to an integrated assessment design. In this understanding, integration includes reflectively positioning the assessment in a context of alternative framing options and with reference to a comprehensive situation analysis (see also Wynne 2003).
- **Anticipation**: The EC specifically mention anticipation as a key element in integration (EC 2011), but also in approaches to responsible research and innovation (RRI) where anticipation has been proposed as essential (see e.g. Von Schomberg 2012 or Owen et al. 2014). In an RRI context assessments should provide the necessary input for responsible governance, and anticipation is then arguably important in an integrated approach.^5^
- **Targeted use of methods in assessment**: The TAMI project (Decker and Ladikas 2004) advocated a comprehensive analysis of assessment purposes and roles in order to make a reflective decision on assessment methodology. In this way a reductive approach to method choice would be avoided.
- **Integration of stakeholders/the public into assessments**: This dimension characterises many current assessment practices that regard themselves as integrated (see. e.g. van der Sluijs and Kloprogge 2010).
- **Integration among assessments**: This kind of integration follows from the definition of integrated assessment given in section 1; namely that existing assessments should be integrated in an overall overview of the issue.
- **Integration of governance concerns into assessment**: This also follows from the definition given in section 1; namely that policy relevant concerns should be integrated into the assessments in order for assessments to properly inform responsible technology policy.
- **Better integration of assessment into governance**: Also mentioned by Scrase and Sheate (2002) this understanding of integration refers to the use of assessment in governance, or more generally, in policy processes.

For our argument, it is not crucial to critically discuss whether all these diverse interpretations are justified, clear or useful. Rather, the given list simply lays out how the initially ambiguous concept of ‘integration’ and ‘integrated assessment’ may be understood in order to identify more specifically what kind of integration is currently observed and what kind of increased integration might potentially be desirable.

### Findings from the analysis of case studies

The eleven integration dimensions (a) to k)) listed in section 3 were used to facilitate reflection on the aspects of integration observed in the four case studies. Because of the diverse nature of the case studies the different dimensions were interpreted in slightly different ways. The dimensions should therefore be regarded as prompts to consider aspects of integration rather than as a clear-cut conceptual grid.

The main findings on integration from the analysis of the case studies are summarised in Table 1:

**Table 1:** Findings on the integration dimensions from the case studies

	Nanotech & Food	Synthetic Biology (SB)	Biofuels	Cloud Computing
a) Inclusion of all areas of topics into assessments	Broader set of topics is already included. More data integration not recommended	A majority of assessments includes a broad set of topics	Social issues lacking in assessments	Many assessments include a broad set of topics but within distinct scientific perspectives
b) Inclusion of values into assessments	Better inclusion of values in assessments is needed	Ethical issues are addressed in the corpus as a whole	Generally lack of explicit values and ethical discussion	Generally low level of reflection on values
c) Inclusion of narratives into assessments	Narratives not included	Not considered much, though some scenarios are addressed	Generally not included	Although hype narratives play a great role in assessments, narratives are not explicated as such
d) Not isolating one topic at the expense of the whole	More topic focused assessments needed taking practical complexity into account	When SB matures and specific applications are developed, this form of integration may become more important	Call for increased consideration of alternatives	Focusing specifically on cloud computing may explain why wider ICT-related issues (e.g. Big Data) are not discussed
e) Explicating assessment framing	Transparency of framing should be increased	Explicit reflection on framing is lacking	Problem framing is generally not clear	Explicit reflection on framing is lacking
f) Anticipation	Systematic anticipation and scrutiny of alternative technology paths is needed	Anticipation is appropriately addressed	Many biofuels assessments are anticipatory	Most assessments have a short-term anticipatory focus but do not investigate longer term implications
g) Targeted use of methods in assessment	In general not much reflection on methods	In general not much reflection on methods	Lack of transparency on methods, in particular concerning Life Cycle Analysis	Some assessments use methods in a business-as-usual manner, others design methods to produce certain types of outcomes
h) Integration of stakeholders/the public into assessments	Less use of participatory approaches over time	Although stakeholder and lay people participation is lacking, how, and to what extent more participation is required is not clear	Much more participation is called for	Very little, more is called for
i) Integration among assessments	More systematic learning is needed	Currently not much integration	An integration institution was called for	The integrating effect is in policy-making, not among the assessments themselves
j) Integration of governance concerns into assessments	Reflection on impacts of governance trends not included in assessments in a systematic way	Not systematically done, though there is reflection on current biotech. governance and regulation and to what extent this suits the (future) field of SB	Governance concerns are well integrated except for the social dimension of sustainability	Due to many assessments being commissioned, in general governance concerns are well integrated in the assessments
k) Better integration of assessments into governance	No information available on how assessments are integrated into governance	Apparently low impact of the assessments on governance	There appears to be a potential better integration, at the expense of consultants	Some assessments seem designed to support policies, not the other way around

The comparison of the analysis of the dimensions in each case study (summarised in Table 1) reveals the following:

- **Inclusion of all areas of topics into assessments:** Substantive integrated assessment approaches are already being developed within the domains.
- **Inclusion of values into assessments**: Though ethical issues are being addressed in the body of assessments as a whole (e.g. in dedicated ethical assessments), there is generally low level of reflection on values in the individual assessments.
- **Inclusion of narratives into assessments**: Narratives are hardly reflected in the assessments.
- **Not isolating one topic at the expense of the whole:** One way to interpret this is to see single technologies as a part of a larger technological field; for instance to write about nanosensors in the context of the development of nanotechnologies in general. Such general assessments are quite frequent, and often of a disciplinary character. Another interpretation of non-isolation is to analyse technologies in rich, problem focused assessments, assessing the consequences of specific technology applications in their complex use situations with their multiple effects. Such highly interdisciplinary assessments were rare.
- **Explicating assessment framing:** The transparency of the framing of the assessments is generally low.
- **Anticipation:** Many assessments have an anticipatory dimension, but few use specific anticipatory techniques.
- **Targeted use of methods in assessment:** Most assessments did not critically discuss the basis and implications of their method choices.
- **Integration of stakeholders/the public into assessments:** There is a varying extent of integration of stakeholders, and a very low extent of integration of the public.
- **Integration among assessments:** In the ICT case study the policy process itself was found to have an integrative effect on the assessments. There were also some integration efforts between assessments; ethical assessments and TA would refer to risk assessments, and impact assessments would refer to economic and environmental assessments. Otherwise there was not much integration across the domains.
- **Integration of governance concerns into assessments:** The integration of governance concerns varied across the case studies, but in general no systematic tools for such inclusion were found.
- **Better integration of assessments into governance:** The impact of assessments on policy is notoriously difficult to investigate (see Decker and Ladikas 2004). Evaluation utilisation studies have developed a sophisticated taxonomy of how evaluations may influence decision making in ways that may be difficult to discern (Herbert 2014), but such analyses were outside the scope of our studies.

As can be seen, the current state of integration in the four technology governance cases varies with the understanding of integration. In some interpretations, like a) inclusion of all areas of topics into assessments, integration is currently well-covered. In other interpretations, like e) explicating assessment framing, there seems to be great room for improvement. However, even if a certain interpretation of integration is currently weakly implemented, it does not follow that there is a need to strengthen it. These normative questions we explored in the literature and in dialogues with stakeholders and end-users in several workshops. The assumption was that the integration analysis in the case studies could inform the knowledge base for making recommendations on integration but that such recommendations would be futile if they did not relate to the assessment practitioners’, policy makers’ and stakeholders’ own experience of challenges in designing, producing and using such assessments for the purposes of responsible science and technology development and governance. Through such dialogues, as well as from a review of literature on EST assessment and governance challenges, some main topics emerged.

## What kind of integration should be strengthened in EST assessment?

### The importance of problem-orientation

Key contributions in the literature on assessment and governance of science and technologies have pointed to the need for solving urgent, complex, real-world problems (Weinberg 1972, Thompson Klein 1990, Decker and Fleischer 2010, Schmidt 2011, Lingner 2011). However, the case studies and domain studies presented here show that integration related to not isolating one topic at the expense of the whole, understood in the sense of rich, problem-oriented assessments (dimension d)), was scarce. On one hand, many of the reviewed assessments are carried out at general levels (addressing issues such as the ethics of synthetic biology or the sustainability of biofuels). Though useful for some purposes this fails to address what a participant in a workshop in 2013 formulated as a main challenge to EST assessment, namely ‘[n]ot to take too much of a bird[‘s eye] view but to really zoom in on the details (without losing focus/broader view) to make the results applicable to ‘daily practice”.^6^ On the other hand, such zooming in cannot be too discipline based if it is to support integrated decision making on policy problems: as another participant noted, current EST assessment practice is strongest when it is interdisciplinary ‘because it forces/challenges you to take a different perspective and critically reflect on your own work’.

To be clear, general assessments mapping out overall issues of concern and specific assessments analysing in-depth specific problem areas are of course crucial to the formation of a policy-supporting knowledge base. However, disciplinary assessments addressing specific aspects of new technologies and their potential use too often fail to provide the necessary bridges from the specific knowledge generated in the assessment and the pragmatic issues of society and policy. Increased integration in the sense of bridging evidence and policy problems would seem to be able to make real progress beyond the state-of-the-art.

Aiming assessments towards problems, and towards specific ways of addressing these, means to provide through the assessment some of that interaction between societal spheres (van Est et al. 2012) that may cause controversy and conflict. To take a problem-oriented approach to integrated assessment means to ground the assessment activity thoroughly in the embedding of science or technology into society. In such situations, issues arise about how techno-scientific development directions align with societal challenges, market trends, political programs and ideologies, and citizens’ wishes and dreams about the future. Knowledge which may seem uncontroversial in one sphere of society enters into a situation of contestation. Assessing the complexity of such a problem may become necessary on the backdrop of an existing societal controversy. However, a problem can also be defined in an anticipatory way; anticipating future problems that should be addressed early.

As the real world is not bound by disciplinary borders, real world problems are necessarily *transdisciplinary*, where the different disciplines need to develop common approaches and where non-scientific competencies are included as important information providers on the practical consequences of issues (see e.g. Boradkar 2012 and Nordmann 2004). In the context of assessment of technologies this means that in many cases several advisory domains should be included in order to appropriately shed light on the issue. This implies a transition from assessments that are rooted in one single advisory domain only to a *trans-domain* approach. Trans-domain problem orientation implies inviting representatives from several domains into a common assessment process. Instead of choosing one privileged domain (for instance impact assessment or technology assessment) where all topics should be integrated, a problem oriented approach seem instead to require that the issue is approached as a cross-cutting learning challenge with implications for all domains. Increased dialogue between assessment communities appeared to be the most important recommendation for integration from the EST-Frame end-users (see Thorstensen et al. 2014, p. 24). However, problem-orientation also implies an acknowledgement of the need to consider the participation of a wider range of actors and interested parties and encourage reflection that transcends technical issues of integration of assessment approaches.

### Transparent assessment framing, method choice and assessment integration

When taking a problem-oriented and trans-domain approach, a cluster of other integration dimensions are implicated. Firstly, this implies a need to integrate state-of-the-art assessments from a variety of domains, corresponding to the dimension of integrating existing assessments (dimension i). The document and literature studies revealed very few studies reviewing the assessment of a technology field in general (a notable exception is the Rathenau study on nanotechnology assessment in the Netherlands, van Est et al. 2012). Overall, the case studies, as well as feedback from end users, indicate that integration between assessments from different domains is a key, unresolved issue. This issue has strong potential implications for policy making and responsible governance of EST because at some point, some kind of integration of the evidence base will be done, in the domain of policy making and politics, in the sense that data or recommendations from assessments are used to inform and justify decisions. In the case studies it was not possible to detect that the selection and use of existing assessments to inform policy decisions was done in a systematic and transparent way. This suggests that an approach that may facilitate transparent integration of lessons from existing assessments would be useful.

Focussing on problem-orientation and trans-domain dialogue also has implications for the relevance of the dimensions concerning explicit assessment framing and method choice (dimensions e) and g)). If problem-orientation, trans-domain interaction and integrating lessons from existing assessments are to be done, the assumptions of the different domain representatives and assessments need to be *transparent*. Assessments with incompatible assumptions may not be possible to integrate. Moreover, the situation analysis and method choice of the integrated process must be explicit and reflective, since there is no privileged perspective from which to frame the issue and assess it (Rein 1976, Stirling 2008). Situation analysis, or scoping (see Stevens 2012), is the first phase of any assessment and ends up in a framing of the assessment.

Similarly, on the methodological side every choice and deployment of assessment method is influenced, though not always explicitly, by fundamental values (see for instance Funtowicz 2006). A wide range of methods can be used in assessments and while the choice of which methods to include in traditional domain-based assessments may be seen as straightforward and disinterested, it plays a decisive role in the results of the assessment process. The importance of explicit and reflective method choice holds in particular for integrated assessment projects where there can be no default assessment methodology in such a diverse assessment group. From the analysis above we saw that the framing of the assessments and the choice of method, is often not explicit. Strenghtening these integration dimensions therefore seems like an important contribution.

In conclusion, from these deliberations there seems to be a need for an integrated approach with the following focus: assessing issues in their complexity as policy problems; facilitating communication between advisory domains, integrating current assessments; and transparent situation analysis and method choice. The four integration dimensions d), e), g) and i) thus appear to be the ones where the need and potential for further development seems to be the greatest. But what about the other integration dimensions? These may for specific issues be important, but do not appear as major unmet needs in EST assessment, according to the end users and the literature studies. An important reason for excluding some of these integration dimension from further development was to avoid increased complexity. Most of the end users wanted a flexible approach and not a strict, multi-dimensional assessment methodology consisting of instructions for anticipation, narrative analysis, etc. Moreover, several integration dimensions were regarded as well-developed, and as such not in need of further development in the EST-Frame context.

Table 2 summarises the resulting prioritisation of integration dimensions for further development.

**Table 2:** Analysis of needs for increased integration

Integration dimensions	Prevalence in case studies	Assigned importance for further development
d) Not isolating one topic at the expense of the whole^a^	Rarely done	High
e) Explicating assessment framing	Rarely done	High
g) Targeted use of methods in assessments	Rarely done	High
i) Integration among assessments	Rarely done	High
f) Anticipation	Varies	Medium
h) Integration of stakeholders/the public	Varies	Medium
j) Integration of governance concerns into assessments	Varies	Medium
b) Inclusion of values into assessments	Rarely done	Medium
c) Inclusion of narratives into assessments	Rarely done	Medium
a) Inclusion of all areas of topics into assessments	Varies	Low
k) Better integration of assessment into governance	Uncertain	Low

^a^Understood as problem-focused analysis

## The Trans Domain Technology Evaluation Process (TranSTEP)

**Fig. 1:**
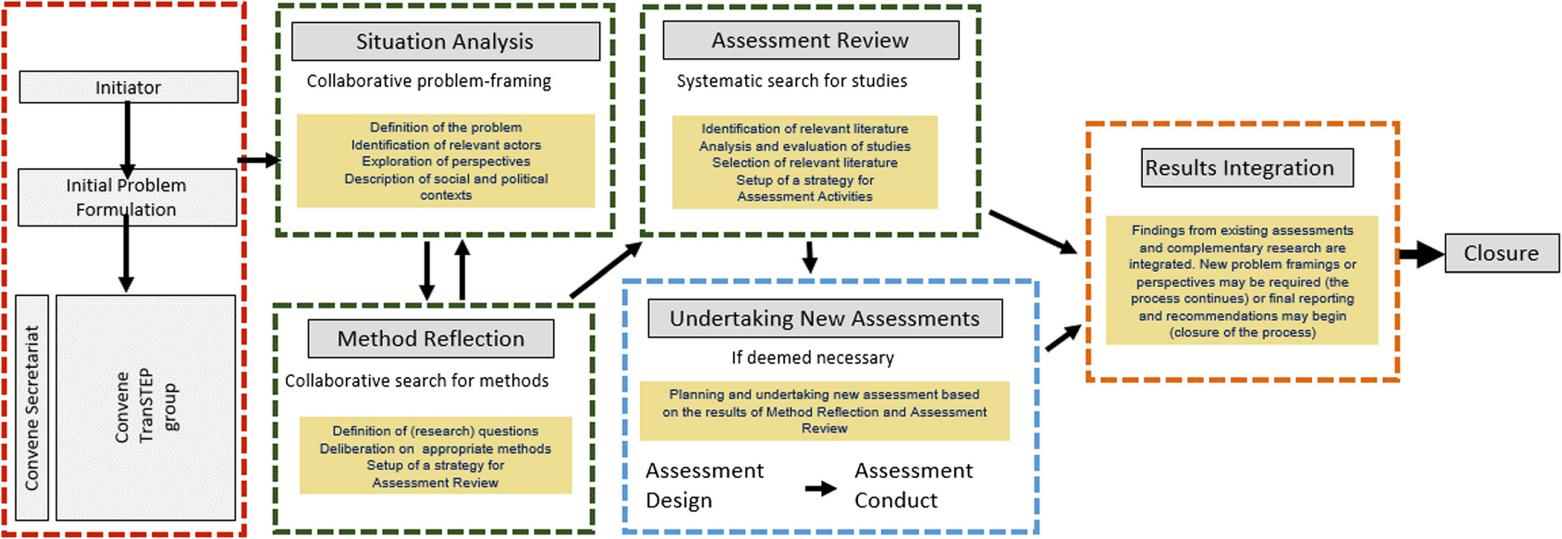
Diagram of the TranSTEP process

Above we have presented integration dimensions that are in need of further development. We believe that addressing the dimensions marked with ‘high priority’ in Table 2 constitutes the greatest progress beyond the state-of-the-art in integrated EST assessment. In the project it was assumed that such integration could be strengthened with a defined approach, assisting practitioners in carrying out such integrated assessments. The so-called TranSTEP approach was thus developed. This integration approach involves organising assessment dialogues across institutional and disciplinary domains; transparent, collaborative situation analysis, problem framing and method reflection; and continual process reflection to adapt to the situation under scrutiny (for details see the webpage http://transtepapproach.wordpress.com/). This includes the previously described key elements, but also includes additional elements considered useful for the approach. We will here spell out in more detail the main elements of TranSTEP^7^ (see Fig. 1).

Using the TranSTEP approach involves initiating and facilitating an assessment group composed of people from different advisory domains, as well as problem owners and other stakeholders, to integrate assessment perspectives on complex technology issues. A TranSTEP group will be convened when a problem owner identifies a specific, complex problem that needs resolution or action. This problem must be given a preliminary definition by the problem owner, allowing for establishing a TranSTEP secretariat that will assist to initially select relevant participants to the trans-domain, or TranSTEP, group. Participants in such processes can be assessment practitioners from domains such as economics, risk assessment, ethics, foresight, impact assessment or technology assessment, or from outside these domains. What domains should be involved will vary from problem to problem. In order to ensure that all relevant perspectives are brought in, problem owners and other actors should also be involved, such as representatives from industry and public research, private sector stakeholders, public sector decision-makers or administrators, NGOs or, if appropriate, the public.

The TranSTEP group will have a preliminary mandate or initial problem formulation from the initiator of the process (problem owner). Situation analysis builds on this initial formulation and it is the role of the TranSTEP group to challenge it and/or elaborate on it in close dialogue with the problem owner. Situation analysis is the first phase of any assessment (even if it is sometimes implicit) and ends up in a framing of the problem that is to be tackled by the assessment. In a trans-domain assessment process, it is particularly necessary to explicate assumptions, purposes and values and place the integrated assessment among them. This includes clearly stating the purpose to be achieved by carrying out an integrated assessment and which role the assessment aims to play. The problem will be further defined, relevant actors will be identified, perspectives and interests explored and the social and political contexts described in detail.

Where participants in a group have to explicate their assumptions, learning about themselves and about their own assumptions related to others’ is inevitable. Moreover, coming to agree on a common assessment framing necessarily involves what van de Poel and Doorn (2013, p. 123) calls ‘reflective learning’. This work is challenging and requires specific process management competencies. Bringing together such a wide range of individuals in a meta-assessment process means that it is likely that they will bring a multitude of implicit situation analyses to the table. The first task in the TranSTEP group is therefore to bring out the assumptions about the situation, critically reflect on them and agree on a common situation analysis and problem understanding that allows the group to work together.

Situation analysis should also include a preliminary reflection on what methods would be appropriate for addressing the problem framed within the group. This is necessary firstly for searching for current and available evidence that may help to address the problem, and secondly if the TranSTEP group decides that new assessments or dialogical activities are needed, since then they will also have to design such new actions. In a TranSTEP group there will be no agreed routine method to be used; the different participants may have different views on what methods would be appropriate. This is a benefit, as it allows for a transparent and reflective method discussion in the group, which in the end may yield more robust judgements than method choices based on implicit conventions or institutional traditions.

Though deliberative situation analysis, problem framing and method choice may sound like a very challenging task for a trans-domain group our experiences from organising four ‘testing workshops’ in the EST-Frame project shows that it is indeed possible. Generally, the participants in these workshops found this work hard, but fruitful.^8^

Once there is a common understanding about what characterises the issue to be assessed and there is agreement upon the problem formulation and upon suitable methods to provide knowledge on the problem, the TranSTEP group, with the assistance of the secretariat, can assess whether existing evidence (previous assessments including deliberative initiatives) can provide the foundation the group needs to conclude on the issue.^9^ The review will end up with a judgement on whether there is sufficient evidence for integrating existing knowledge into a conclusion on the problem. If the group believes that it is not, then new assessment activities will, if possible, be initiated by the group.

The group may have resources to undertake such actions themselves (such as organising a citizen’s panel) or the group may encourage relevant problem owners or stakeholders to organise such an assessment (for instance an impact assessment). In any case, the group must engage in detailed reflection on the required methods. As noted above, a wide range of methods can be used in assessments and reflecting on the strengths and weaknesses of this broad range of methods for tackling the agreed assessment problem is therefore necessary. Several former and ongoing European projects, such as DoingForesight, Sustainability A Test and Engage 2020, provide overviews of tools to make up a comprehensive tool box. These can be used to raise awareness of the wide range of method available to provide evidence for the problem solution, so that such choices are made in a reflected and transparent manner and not simply in an intuitive, implicit way resorting to default methods that might not fit the sophisticated situation analysis developed in the TranSTEP group.

Note that potential new assessment activities do not necessarily need to apply inter- or trans-disciplinary methods. What is needed may be (for instance) a traditional risk assessment, ethical assessment or foresight, if this is the knowledge lacking for an integrated conclusion in the TranSTEP group. New assessment activities can, but do not necessarily have to, be undertaken by the TranSTEP group (assisted by the secretariat). However, if outsourced, the TranSTEP group should be involved in or regularly informed about the new assessment activities and outcomes.

By drawing lessons from earlier assessments and initiating new assessments/deliberative events to fill knowledge gaps (including clarifying the extent of uncertainties that will have to be addressed by decision makers), the TranSTEP group will produce integrated conclusions to support the creation of responsible policies for research and innovation. If the group cannot initiate new assessment activities it will integrate the review into a statement of the current knowledge status, with recommendations for further assessment activities to be initiated by other relevant actors.

The TranSTEP group will decide to end the process when a) they believe there is sufficient evidence (on facts, values, perceptions or alternatives) for concluding on the issue they have defined; or b) when practical constraints (such as available funding) make it impossible to continue. At this point a report will be written integrating the results and deliberations of the process. Results integration is a matter of collective judgement in the TranSTEP group. No algorithm can be provided, only argumentation based on the preceding steps. Integration of the results will take the lessons from previous and, potentially, new assessments and apply them to the problem formulation, allowing a judgement to be taken on each aspect of the problem formulation. As such the integrated assessment will be both a meta-assessment, in the sense that it integrates the current assessment knowledge base, and a new transdisciplinary assessment. Depending on the reviewed evidence and the problem formulation the group may end up with a consensus on recommendations regarding specific decisions or policies or mapping of points of consensus and dissent. Even if the group does not end up with an agreement, reporting the situation analysis, assessment design deliberations and the type of dissent will still be of great value to policy makers and other decision makers.

It should be noted that even if Fig. 1 indicates a procedure with a clear direction, there might in reality be a need for revisiting previous stages as the group’s understanding of the issues develops. New insights might reveal the need for adjusting the initial situation analysis and framing of the issues. The procedure depicted in Fig. 1 is not intended to limit such reflective iteration. Being open to adjusting the process to new circumstances or new perspectives is an important condition for the assessment of emerging technologies in situations of complexity and uncertainty.

Moreover, it should be clear that the robustness of the conclusions of the process will depend upon the quality of the deliberations. The conclusions will mirror the process of deliberation and will be a contribution to the knowledge on the issue at hand that reflects the knowledge status at the time of integration and the composition of the TranSTEP group.^10^

Finally, it should be mentioned that transparency is a fundamental condition for the work in an integrated assessment process. Transparency involves being open about all issues of public interest: the situation analysis (including the problem framing), the justification of the method choices, the assessment reviews and the contested versus undisputed points of the dialogue process. Transparency is crucial in assessments that aim to give substantial advice and concrete recommendations but also in assessments that aim to explore issues in a more open fashion (see Stirling 2008) and is of particular importance of integrated assessments where the procedures by definition extends beyond the established, and often documented, conventions in the individuals domains. Revealing thoroughly the limitations and assumptions of the integrated assessment means to reveal fully the assessment as an act carried out in a specific time and place and to allow recipients to take this into account in their own reflections. By revealing the limitations of the assessment, the nature of the subsequent use of the results by others can become transparent in turn. But even if transparency is important for the legitimacy of the integrated assessment, it needs to be balanced with the need for a protected space for open dialogue.^11^

## Discussion

Methodological issues of the case study and domain study research have been discussed in the articles referred to above (Forsberg et al. 2014, De Bakker et al. 2014, van Doren and Heyen 2014, van Doren et al. 2014, and Boucher et al. 2014). Although the integration analyses presented here might suffer from a certain degree of conceptual ambiguity and differing interpretations in the different case studies, the overall diagnosis of the state of integration in the four case studies are confirmed by key end-users.

It should be noted, though, that the empirical work presented here is mostly limited to the four case studies. It is possible that we would have other findings if we analysed the assessment of nano food in the UK or cloud computing in Germany. However, the analyses of advisory domains and discussions with end-users give no indication of any systematic bias resulting from the selection of case studies. Still, we believe that the next step in the research is to analyse a broader range of case studies.

The bottom-up process leading to the TranSTEP approach has had the effect that the outcome is not novel, but based on experiences of already existing practices. It must readily be admitted that similar ideas and approaches as the ones presented here have been launched before, for instance the PRIMA approach in the Dutch National Institute for Public Health and the Environment (Van Asselt et al. 2001) and the approach of the European Academy of Technology and Innovation Assessment, and in conceptual work by Tribe (1973), Schön and Rein (1994) and others referred to above. However, earlier contributions have failed to highlight the importance of targeting learning between assessment communities. This is an important point because governance of new technologies depends in great part on assessment in institutionalised communities, in particular risk assessment and economic assessment communities, and in some cases TA, ethics committees, foresight and impact assessment communities. Sometimes this work provides a transparent basis for decisions, as when risk assessment institutions assess the risk according to a regulatory framework. Other times the assessments are less transparent in their design and in the way they influence policy making. The EST-Frame end-user workshops confirmed that the assessment communities face the same challenges, especially related to dealing with uncertainty and tackling public controversy.

Moreover, advisory domains are in some cases interconnected by dependence. Uncertainties in risk assessment will often result in even larger uncertainties in the subsequent economic assessments (see for instance the common report of the EC Scientific Committees: SCCS, SCENIHR & SCHER 2013, p. 8). And decisions on risk parameters have value-dimensions that ethical committees may see as their business (see for instance WHO 2002). However, these communities – even if they recognise these dependencies (e,g, the Paris Risk Group^12^) – seldom engage with each other in practice. There might be several reasons for this; and one important reason is probably connected to the institutionalisation of the work in these domains. In order to increase integration so-called double loop learning (Schön and Rein 1994) needs to be stimulated so that the assumptions in the domains are challenged. Trans-domain assessment approaches have the potential to do this because cooperation on assessments engage domain practitioners in reflections on their own assumptions that can be a stimulus for reflections also internally in their ‘home’ communities. This can subsequently lead to domain assessments that themselves are more reflective.

Sarewitz (2010) have argued that institutional reform is needed to address the challenges for the assessment of emerging science and technologies in situations where facts are uncertain, values are disputed, stakes are high and decisions are urgent. The trans-domain nature of TranSTEP is an argument against establishing such integrated assessments as a new institution or domain in itself. The TranSTEP approach may instead be seen as an institutional innovation that continues to make each trans-domain assessment process an innovation that is adapted to the specifics of the context in which it is used. This implied institutional reform is neither hard in the sense of calling for a novel space for trans-domain assessment nor radical in the sense of abandoning existing assessment traditions, but institutional reform nevertheless.

A participant at one of the final workshops asked: “Is it revolutionary? Yes, perhaps, because the concept might consider different framings from different problem owners and therefore might be able to internalise plurality and different perspectives, which is powerful.” As such, the revolutionary aspect of trans-domain integration is not in its concepts, but in the way it might be used. Different frameworks and approaches may achieve such revolutionary effects, and TranSTEP is just one approach. The work presented here points to two strategies for further work; increased experimentation with trans-domain assessment and further research on such experiments and on the learning processes between assessors and between assessor and policy-makers.

## Conclusion

We have here mapped out integration dimensions for EST assessment and showed how these are currently addressed in four case studies. Needs for increased integration have been discussed with reference to key contributions in the literature and discussion with end-users. From this we induced the need for an integrated approach for assessing issues in their complexity as policy problems, facilitating communication between advisory domains and integrating current assessments by applying transparent situation analysis and method choice. We have outlined these dimensions in some detail and suggested how they can be addressed in a practical process.

By taking the analytical route presented above, we have avoided two pitfalls. On the one hand, the research approach has avoided taking one given conception of ‘integrated assessment’ as authoritative, allowing instead the full range of existing contributions to the diverse field of integrative assessment to function as a reservoir for solutions to real-world quality issues in EST assessment. On the other hand, the research has also avoided the path leading towards a kind of ‘super’-assessment, which would seek to synthesise all types of ‘integration’ into a unified approach. Instead, we have sought to provide an assessment framework able to supplement and make use of existing assessment approaches so as to increase the usefulness of assessment work in general for decision-making in areas of uncertainty and contestation.

While this proposal is consistent with certain methodological proposals from the TA and sustainability assessment domains, it goes beyond these domain-specific frameworks by taking a trans-domain approach. As such, TranSTEP is integration for professionals, aiming to impact on professional practices. As a transdisciplinary and reflective approach, it is consistent with the increasing focus on RRI (Forsberg et al. 2015 and Ribeiro et al. 2016). It is useful for assessment professionals who wish to position their assessments better in the environment outside their own institutions and it is important as a learning process within and between assessment communities in the longer term. Finally, the trans-domain feature is crucial for decision makers and policy makers that need to align and balance advice from different advisory domains.

## Footnotes

1 With the term ‘advisory domain’ we refer to institutionalised assessment traditions such as economic assessment, risk assessment, impact assessment, foresight, ethical assessment and TA. Conceptually, we have abstained from attempts at building a strong epistemology for the concept of ‘domains’. The point has been to delimit our selection of domains from emerging or hybrid forms of assessment taking place as one-off experiments or transient phenomena and to focus instead on what may be said to be well-known interfaces between science, society and policy (see also Forsberg et al. 2014). With the idea of institutional domains comes some degree of tradition, some common forms of practice and some degree of establishment within public decision-making systems.

2 http://ec.europa.eu/smart-regulation/impact/background/background_en.htm [Last accessed 01.01.16].

3 http://www.who.int/ipcs/methods/risk_assessment/en/index.html [Last accessed 01.01.16].

4 http://estframe.net/.

5 Initially we opened up for the possibility that a range of methodological elements might be included here and that anticipation should be seen as one suggestion. However, it turned out that we did not find any other element (other than the ones mentioned in the other dimensions on this list) that was called for in the context of integrated assessment, so anticipation became the focus of this dimension.

6 Quoted from the feedback form.

7 The website provides more detail and optional resources for assessment groups that would like more specific guidance in their integrated assessment.

8 Since these workshops only tested this first part of the integrated assessment approach the scores on the feedback forms indicate that this was considered useful: The average score on the statement ‘There is a need for an integrative process such as the Integrated EST framework in this field’ was in the synthetic biology workshop 4,2 (on a scale from 1 to 5 where 1 was ‘completely disagree’ and 5 was ‘completely agree’). The average score on ‘There is a need for an integrative process such as the Integrated EST framework in other fields’ was in the same workshop 4,1. The scores were identical in the biofuels workshop. In the cloud computing workshop the scores were, respectively, 3,7 and 4,3. In the nano food workshop the average scores were, respectively, 4.3 and 4.1. In total, 45 external participants attended these workshops.

9 The EST-Frame analytic protocols may prove helpful for carrying out this task.

10 Wider hearings of draft conclusions of the TranSTEP group might be advisable, so that potentially neglected perspectives may be included.

11 On the TranSTEP website guidelines are provided for such balancing.

12 https://parisriskgroup.anses.fr/.

## References

[CR1] Bond A, Morrison-Saunders A, Pope J (2012). Sustainability assessment: the state of the art. Impact Assessment and Project Appraisal.

[CR2] Boradkar P. ‘Design as problem solving’ In: The Oxford Handbook of Interdisciplinarity, Frodeman, R., Thompson Klein, J., Mitcham, K. Oxford University Press; 2012, p. 273–87

[CR3] Boucher P, Smith R, Millar K (2014). Biofuels under the spotlight: The state of assessment and potential for integration. Science and Public Policy.

[CR4] Cohen S, Neale T (2006). Participatory Integrated Assessment of Water Management and Climate Change in the Okanagan Basin, British Columbia.

[CR5] Davies S, Macnaghten P, Kearnes M (2009). Reconfiguring Responsibility: Lessons for Public Policy (Part 1 of the report on Deepening Debate on Nanotechnology).

[CR6] de Bakker E, de Lauwere C, Hoes A-C, Beekman V (2014). Responsible research' and innovation in miniature: Information asymmetries hindering a more inclusive ‘nanofood‘ development. Science and Public Policy.

[CR7] de Ridder W, Turnpenny J, Nilsson M, von Raggamby A (2007). Framework for Tool Selection and Use in Integrated Assessment for Sustainable Development. Journal of Environmental Assessment Policy and Management.

[CR8] Decker M, Fleischer T (2010). When should there be which kind of technology assessment? A plea for a strictly problem-oriented approach from the very outset. Poiesis and Praxis.

[CR9] Decker M, Ladikas M (2004). Bridges between science, society and policy: technology assessment - methods and impacts.

[CR10] Epstein JM (1999). Agent-Based Computational Models and Generative Social Science. Complexity.

[CR11] European Commission (2010). Work programme 2011, Capacities, part 5, Science and Society.

[CR12] European Commission. Emerging Science and Technology priorities in public research policies in the EU, the US and Japan. 2006 ftp://ftp.cordis.europa.eu/pub/foresight/docs/ntw_emerging_report_en.pdf [Accessed 20.10.2014]

[CR13] Forsberg E-M, de Lauwere C (2012). Integration needs in assessments of nanotechnology in food and agriculture. Nordic Journal of Applied Ethics.

[CR14] Forsberg E-M, Thorstensen E, Nielsen RØ, de Bakker E (2014). Assessments of emerging science and technologies: mapping the landscape. Science and Public Policy.

[CR15] Forsberg E-M, Quaglio G, O’Kane H, Karapiperis T, Van Woensel L, Arnaldi S (2015). Assessment of science and technologies: Advising for and with responsibility. Technology in Society.

[CR16] Funtowicz SO, Guimaraes Pereira A, Guedes Vaz S, Tognetti S (2006). Why knowledge assessment?. Interfaces between science and society.

[CR17] Guston D, Sarewitz D (2002). Real-time technology assessment. Technology in Society.

[CR18] Hare M, Deadman P (2004). Further towards a taxonomy of agent-based simulation models in environmental management. Mathematics and Computers in Simulation.

[CR19] Herbert JL (2014). Researching Evaluation Influence: A Review of the Literature. Evaluation Review.

[CR20] Jasanoff S (2003). (No?) Accounting for expertise. Science and Public Policy.

[CR21] Joss S, Bellucci S (2002). Participatory technology assessment: European perspectives.

[CR22] Lingner S (2011). Science, interdisciplinarity and the society. Poiesis and Praxis.

[CR23] Mongin P (2006). Value Judgments and Value Neutrality in Economics. Economica.

[CR24] Nordmann A. Converging Technologies. Shaping the Future of European Societies. A Report from the High Level Expert Group on “Foresighting the New Technology Wave”. 2004 http://bookshop.europa.eu/en/converging-technologies-pbKINA21357/ [Accessed 01.05.2013]

[CR25] Owen R, Macnaghten P, Stilgoe J (2014). Responsible research and innovation: From science in society to science for society, with society. Science and Public Policy.

[CR26] Rein M (1976). Social Science and Public Policy.

[CR27] Ribeiro B, Smith R, Millar K. A Mobilising Concept? Unpacking Academic Representations of Responsible Research and Innovation. Science and Engineering Ethics. 2016 http://link.springer.com/article/10.1007%2Fs11948-016-9761-6. 10.1007/s11948-016-9761-6 26956121

[CR28] Robinson DKR, Huang L, Guo Y, Porter AL (2013). Forecasting Innovation Pathways (FIP) for new and emerging science and technologies. Technology Forecasting & Social Change.

[CR29] Rotmans J, Dowlatabadi H, Rayner S, Malone EL (1998). Integrated Assessment of Climate Change: Evaluation of Methods and Strategies. Human Choices and Climate Change: A State of the Art Report, vol. 3.

[CR30] Sarewitz D. Against Holism. In: The Oxford Handbook of Interdisciplinarity*.* ed. Frodeman, R., Thompson Klein, J. and Mitcham, K. Oxford University Press; 2010. p. 65–78

[CR31] SCCS, SCENIHR & SCHER. 2013. Making Risk Assessment More Relevant for Risk Management. http://ec.europa.eu/health/scientific_committees/consumer_safety/docs/sccs_o_130.pdf [Accessed 25.03.15]

[CR32] Schmidt J (2011). What is a problem?. On problem-oriented interdisciplinarity, Poiesis and Praxis.

[CR33] Schön D, Rein M (1994). Frame Reflection. Toward the Resolution of Intractable Policy Controversies.

[CR34] Schot J, Rip A (1997). The Past and Future of Constructive Technology Assessment. Technology Forecasting and Social Change.

[CR35] Scrase JI, Sheate WR (2002). Integration and integrated approaches to assessment: What do they man for the environment?. Journal of Environmental Policy & Planning.

[CR36] Smith T (2010). Using critical systems thinking to foster an integrated approach to sustainability: a proposal for development practitioners. Environment, development and sustainability.

[CR37] Soncini-Sessa R, Castelletti A, Weber E (2007). Integrated and Participatory Water Resources Management, Practice.

[CR38] Stevens C. A Basic Roadmap for Sustainability Assessments: The SIMPLE Methodology. In: Rubik Sustainable Development, Evaluation and Policy-Making. Theory, Practice and Quality Assurance. ed. Von Raggamby, A. and F. Edward Elgar. 2012; p. 57–72

[CR39] Stirling A (2008). “Opening up“ and “closing down“: Power, Participation, and Pluralism in the Social Appraisal of Technology. Science, Technology and Human Values.

[CR40] Thompson Klein J (1990). Interdisciplinarity: history, theory, and practice.

[CR41] Thorstensen E, Forsberg E-M, van Doren D, Heyen N, Reiss T, de Bakker E, Nielsen RØ, Ribeiro B, Smith R, Millar K. EST-Frame Deliverable 6.7 An integrated framework for assessing societal impacts of emerging science and technologies. 2014 http://estframe.net/publications/content_1/text_721891ce-f43b-460e-80ed-339c02c7134d/1418825021825/estframe_deliverable_6_7_final.pdf [Accessed 01.01.16]

[CR42] Tribe L (1973). Technology Assessment and the Fourth Discontinuity: The Limits of Instrumental Rationality. Southern California Law Review.

[CR43] Van Asselt MA, Rotmans J, Greeuw SCH (2001). Puzzle-Solving for Policy: A provisional handbook for Integrated Assessment.

[CR44] Van de Poel I, Doorn N. Ethical parallel research: A Network Approach for Moral Evaluation (NAME). In: Doorn, N., Schuurbiers, D., van de Poel, I. and Gorman, M.E. (eds.) Early Engagement and New Technologies: Opening up the Laboratory. Springer. 2013.; p. 111–136

[CR45] Van den Ende J, Mulder K, Knot M, Moors E, Vergragt P (1998). Traditional and Modern Technology Assessment: Toward a Toolkit. Technology Forecasting and Social Change.

[CR46] Van der Sluijs J, Munn RE, Tolba M (2002). Integrated Assessment. Encyclopaedia of Global Environmental Change.

[CR47] Van der Sluijs J, Kloprogge P, Decker M (2010). The Inclusion of Stakeholder Perspectives in Integrated Assessment of Climate Change. Interdisciplinarity in Technology Assessment. Implementation and its Chances and Limits.

[CR48] Van Doren D, Heyen NB (2014). Synthetic biology: Too early for assessments? A review of synthetic biology assessments in Germany. Science and Public Policy.

[CR49] Van Doren D, Forsberg E-M, Lindner R (2014). Are assessments responding to a dynamic environment? Evidence from four emerging techno-scientific domains. Science and Public Policy.

[CR50] Van Est R, Walhout B, Rerimassie V, Stemerding D, Hansen L (2012). Governance of Nanotechnology in the Netherlands - Informing and Engaging in Different Social Spheres. International Journal of Emerging Technologies and Society.

[CR51] Von Schomberg R. ‘Prospects for technology assessment in a framework of responsible research and innovation’. In: Technikfolgen Abschatzen Lehren. Bildungspotenziale Transdisziplinarer Methoden (Eds. Dusseldorp, M. and Beecroft, R.). Vs Verlag Fur Sozialwissenschaften. 2012;, 39–61.

[CR52] Weinberg AM (1972). Science and Trans-Science. Minerva.

[CR53] WHO (2002). The World Health Report: Reducing Risks, Promoting Healthy Life.

[CR54] Wynne B (1992). Uncertainty and environmental learning: reconceiving science and policy in the preventive paradigm. Global Environmental Change.

[CR55] Wynne B (2003). Seasick on the Third Wave? Subverting the Hegemony of Propositionalism. Response to Collins and Evans (2002). Social Studies of Science.

